# Multiple modes of data sharing can facilitate secondary use of sensitive health data for research

**DOI:** 10.1136/bmjgh-2023-013092

**Published:** 2023-10-06

**Authors:** Tsaone Tamuhla, Eddie T Lulamba, Themba Mutemaringa, Nicki Tiffin

**Affiliations:** 1 South African National Bioinformatics Institute, University of the Western Cape, Bellville, South Africa; 2 Provincial Health Data Centre, Health Intelligence Directorate, Western Cape Department of Health and Wellness, Cape Town, Western Cape, South Africa; 3 Computational Biology Division, Department of Integrative Biomedical Sciences, University of Cape Town, Rondebosch, Western Cape, South Africa

**Keywords:** Public Health, Health policies and all other topics

## Abstract

Evidence-based healthcare relies on health data from diverse sources to inform decision-making across different domains, including disease prevention, aetiology, diagnostics, therapeutics and prognosis. Increasing volumes of highly granular data provide opportunities to leverage the evidence base, with growing recognition that health data are highly sensitive and onward research use may create privacy issues for individuals providing data. Concerns are heightened for data without explicit informed consent for secondary research use. Additionally, researchers—especially from under-resourced environments and the global South—may wish to participate in onward analysis of resources they collected or retain oversight of onward use to ensure ethical constraints are respected. Different data-sharing approaches may be adopted according to data sensitivity and secondary use restrictions, moving beyond the traditional Open Access model of unidirectional data transfer from generator to secondary user. We describe collaborative data sharing, facilitating research by combining datasets and undertaking meta-analysis involving collaborating partners; federated data analysis, where partners undertake synchronous, harmonised analyses on their independent datasets and then combine their results in a coauthored report, and trusted research environments where data are analysed in a controlled environment and only aggregate results are exported. We review how deidentification and anonymisation methods, including data perturbation, can reduce risks specifically associated with health data secondary use. In addition, we present an innovative modularised approach for building data sharing agreements incorporating a more nuanced approach to data sharing to protect privacy, and provide a framework for building the agreements for each of these data-sharing scenarios.

**Summary box**

- Data sharing can ensure maximal ethical use of data resources to inform evidence-based health care.
- Different models of data sharing that move beyond direct open access sharing can be used to address challenges arising from ethical and equity constraints on data re-use.
- Data anonymisation and perturbation can increase protection of privacy and data security for sensitive data.
- A framework using modularised data sharing elements can facilitate creating fit-for-purpose data sharing agreements.

## Introduction

 Data about health is the fundamental base on which evidence-based healthcare is constructed and underpins progress and innovation in health sciences and healthcare towards improved patient outcomes.^1^,^3^ Traditionally, however, competitive research practices have discouraged data sharing,^4^ and researchers may withhold research datasets they have generated in order to protect their career interests and retain the capacity to publish innovative and high impact research. In addition, concerns about intellectual property (IP) and commercial applications have also created barriers to secondary use of health data beyond the primary purpose for which they were collected.^5^

There has been a growing recognition of the need to use and re-use health data for as many diverse and future analyses as possible, within the scope of permissions for use provided by research participants through their informed consent. This need reflects an ethical imperative to maximise the benefits for evidence-based healthcare from the use of health data as an offset against the risks or discomfits faced by participants contributing those data,^6 7^ and has led to increasing pressure on researchers to make data and research outputs Open^8 9^ meaning that access to scientific resources should be unrestricted and free of charge wherever this is possible. The prioritisation of Open Access has resulted in requirements to adhere to Open data access principles in order to receive research funding and to publish research findings in academic journals.^10^ The Open Access principles are also reflected in FAIR principles, which provide guidelines for ensuring data and resources are Findable, Accessible, Interoperable and Reusable.^11^

While there is widespread support for the Open principles, more recently it has become evident that not all data—and especially sensitive personal data such as health or genomic data—are appropriate for Open Access and unrestricted re-use. Furthermore, depending on the national privacy legislation and Health Act in place, reusing certain kinds of sensitive data without participant consent or moving these data across borders may be illegal. Not all data can be made Open in line with Open Access principles, for example some large datasets and real-world data are generated without informed consent from individuals and yet are granular enough to potentially be used to reidentify individuals if combined with other identified data resources.^12^ A prominent example is the use of anonymised health datasets generated from routine health data or electronic medical records, which carry the potential risk of reidentification of health clients who have not consented to participate in research, have not been informed of the risk of reidentification, and have not had an opportunity to choose whether their sensitive health data are used for research.^13^ The epidemiological and health systems value of mining these data is undisputed, but the risks posed to unwitting participants should be absolutely minimised given the circumstances under which the data are generated and used.

Another scenario in which Open secondary use cannot always be implemented occurs with legacy datasets which were collected in the past at a time when legislation and general research practices were much more permissive about data collection without detailed informed consent, during which time uniquely identifying data such as genomic data may have been generated without the knowledge or agreement of those individuals or without their explicit consent for secondary data use. These data cannot ethically be handed on to additional researchers without participant informed consent for secondary use, as this would expose participants through the use of their data to associated risks—to which they have not agreed.

With the rapid growth of genomic data generated from global populations there is increasing recognition of the potential for additional family and community harms that might arise from the analysis of these data.^14^ Although individual informed consent might be in place, consideration must equally be given to the risks of onward data use for relatives, associated communities and identifiable population groups. If an individual’s genomic data are Open and identifiable, what might be the implications for their offspring or relatives who did not consent to the use of those data? How are communities affected when their population-level genetic or epidemiological data become open information, for example stigma that might arise when high risk genetic variants or particular diseases are associated with a specific population group? The community-level impact of genomic studies with San participants in Southern Africa clearly illustrates these risks.^15 16^ Responsible data governance, sharing, analysis and reporting are particularly important to support the inclusion of underrepresented populations in health research, in order to ensure that innovations and new therapeutic approaches are equitable and effective for all populations groups; and equitable and appropriate sharing of data from under-represented groups can contribute to addressing the existing bias in health research.^17^,^19^

Fortunately, together with the growing availability of granular and identifying datasets and a concomitant growing recognition of the need to protect the interests of individuals, communities and researchers, there has been rapid growth in the development of data governance and ethical data use to address these challenges. Traditionally, Open data sharing has been viewed as a unidirectional process whereby researchers who collect and generate data pass them onward for secondary use, either directly or via centralised repositories. In the process they must usually cede any control over how the data are used further. Recently, more nuanced approaches are being developed to ensure maximal ethical secondary use of data resources while minimising risks and respecting the level of informed consent provided by participants.

Here, we provide an overview of four different approaches to data sharing that can be adopted according to data sensitivity and/or restrictions on secondary use. We discuss direct data sharing in a traditional model; collaborative data sharing which facilitates research by combining datasets and undertaking meta-analysis involving all collaborating partners; federated data analysis, in which partners undertake synchronous and harmonised analyses on their independent datasets and then combine the results of their analyses in a final coauthored report; and the use of trusted research environments (TREs) in which data may be analysed in a controlled environment from which only aggregate research results may be exported. We also review how deidentification and anonymisation methods can reduce the risks associated with secondary use of health data. In addition, we present a modularised approach for building data-sharing agreements (DSAs), with a framework that can be used to build such an agreement for each of these scenarios. This provides a new approach to building such agreements that is accessible and manageable for researchers without prior experience in drafting such memoranda.

We have focused here primarily on sharing of data which have been generated directly from individuals and/or biospecimens collected from individual participants. Many of the principles we outline here can similarly be applied to secondary use of biospecimen collections, and while the re-use of biospecimens is not covered exhaustively we have noted some areas where these data-sharing principles may also relate to the sharing of biospecimens.

## Modes of data sharing

Here we discuss four modes of secondary data sharing that may accommodate some of the challenges associated with sharing data and conducting meta-analyses, as outlined in table 1.

**Table 1:** Key advantages and challenges for different modes of secondary data sharing

Mode of sharing	Advantages	Challenges
Direct sharing	Promotes wide re-use and repurposing of data and biospecimens for new insights.	Ensuring equitable agreements and negotiating benefit sharing is difficult, there is no oversight of onward use of resources.
Collaborative analysis	Generates large datasets with statistical power to make new inferences; allow oversight by data generators ensuring ethical onward use of data.	Collaborations can be difficult to set up; consensus may be tricky for authorship roles, attribution; IP may be difficult to assign.Data may be difficult to harmonise and combine.
Federated analysis	Can make use of resources that might not otherwise be shared due to incomplete consent or sensitive data.	Cross-dataset validation may be difficult; consensus may be tricky for authorship roles, attribution; IP may be difficult to assign.
Trusted research ecosystem	Sensitive data are not exposed and cannot be inappropriately shared or used.	Significant investments are required to set up and maintain infrastructure, governance and oversight of TREs.

IP, intellectual property; TRE, trusted research environment.

### Direct sharing

**Figure 1:**
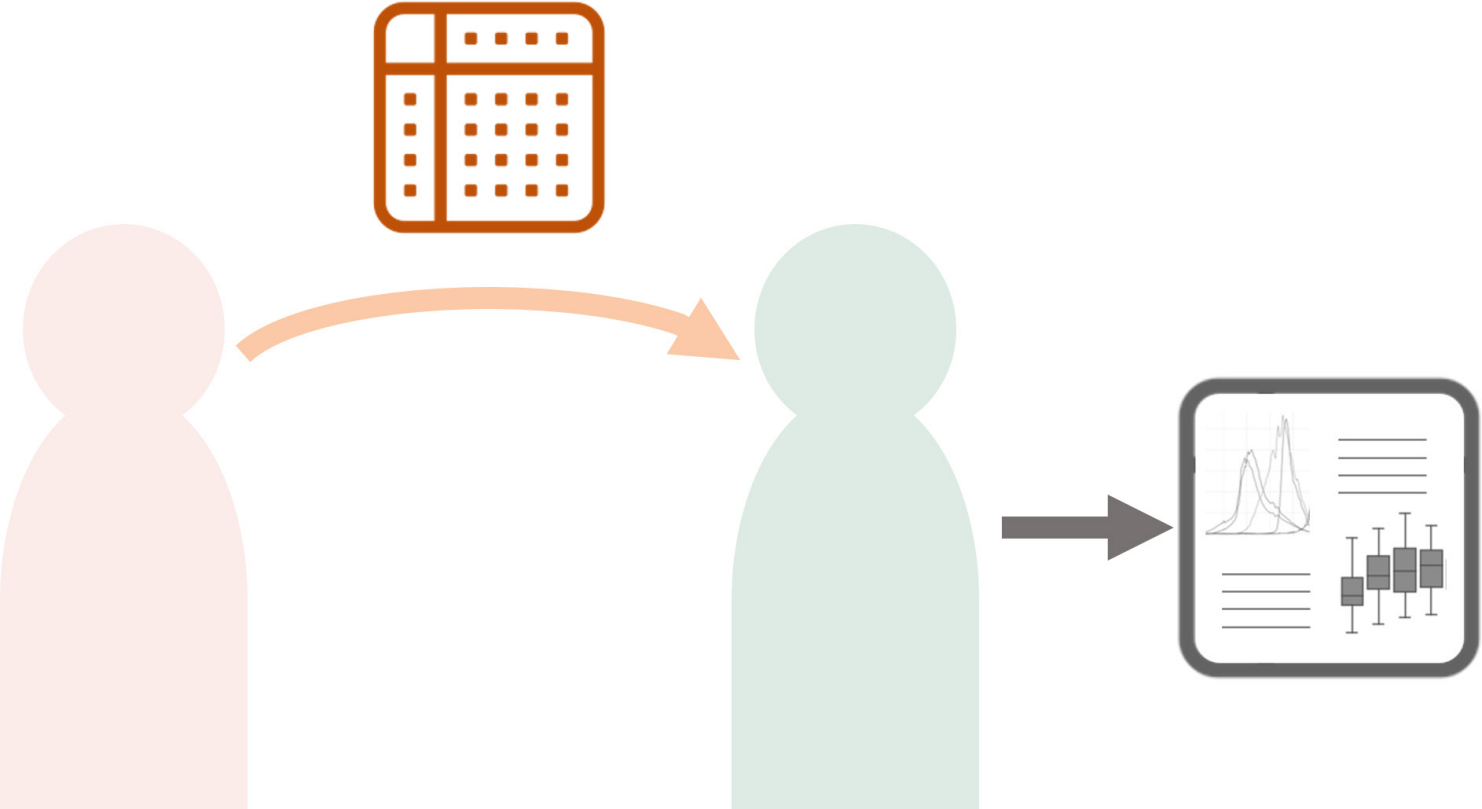
Direct sharing. Unidirectional transfer of resources from generator to consumer. The consumer performs data analysis and generates the research output.

Direct data sharing is the traditional model of data sharing which has been most commonly in use and is routinely required by funders and peer-reviewed journals. In this model, the researcher who has generated data, the data producer, provides a full set of the data to other users, data consumers, for all types of secondary use (figure 1). This may be done via a specific centralised repository—for example, the H3Africa programme (www.h3africa.org) funders require submission of genomic data from the programme to the European Genome-Phenome Archive (https://ega-archive.org/) and submission of biospecimens to centralised H3Africa biorepositories.^20^ Controlled access for secondary use occurs under the oversight of a Data and Biospecimen Access Committee,^21^ and an embargo period formalises a time period for which the data generators have protected access to the data for analysis and publication to ensure they are not scooped by secondary users. Another example is the Research Resource for Complex Physiologic Signals (PhysioNet, https://physionet.org/about/^22^), which offers free access to large collections of physiological and clinical data, while facilitating a level of control over the data by resource generators and promoting collaboration and data sharing.

A central challenge for direct sharing of health data is how to ensure that the privacy of participants is maintained.^23^ While it remains impossible to anonymise genomic data, which is by its intrinsic nature identifying,^24^,^26^ participant safe-guarding for the use of highly granular clinical and epidemiological data can be achieved through data deidentification, anonymisation and also data perturbation. As large datasets become increasingly granular and health metrics become increasingly precise, the opportunity to reidentify individuals through cross-reference with other records does become higher—for example, given the dates and locations of sequential health facility visits together with participant age and comorbidity profile it becomes feasible to reidentify a study participant through cross reference to identified routine health facility records. Legislation that protects privacy increasingly recognises the sensitivity of health data and may offer specific protections in addition to national Health Acts that enshrine healthcare client confidentiality. An example of this is the Protection of Personal Information Act in South Africa which categorises health data as ‘special’ data, requiring additional considerations and protections.^27^

### Collaborative meta-analysis

**Figure 2:**
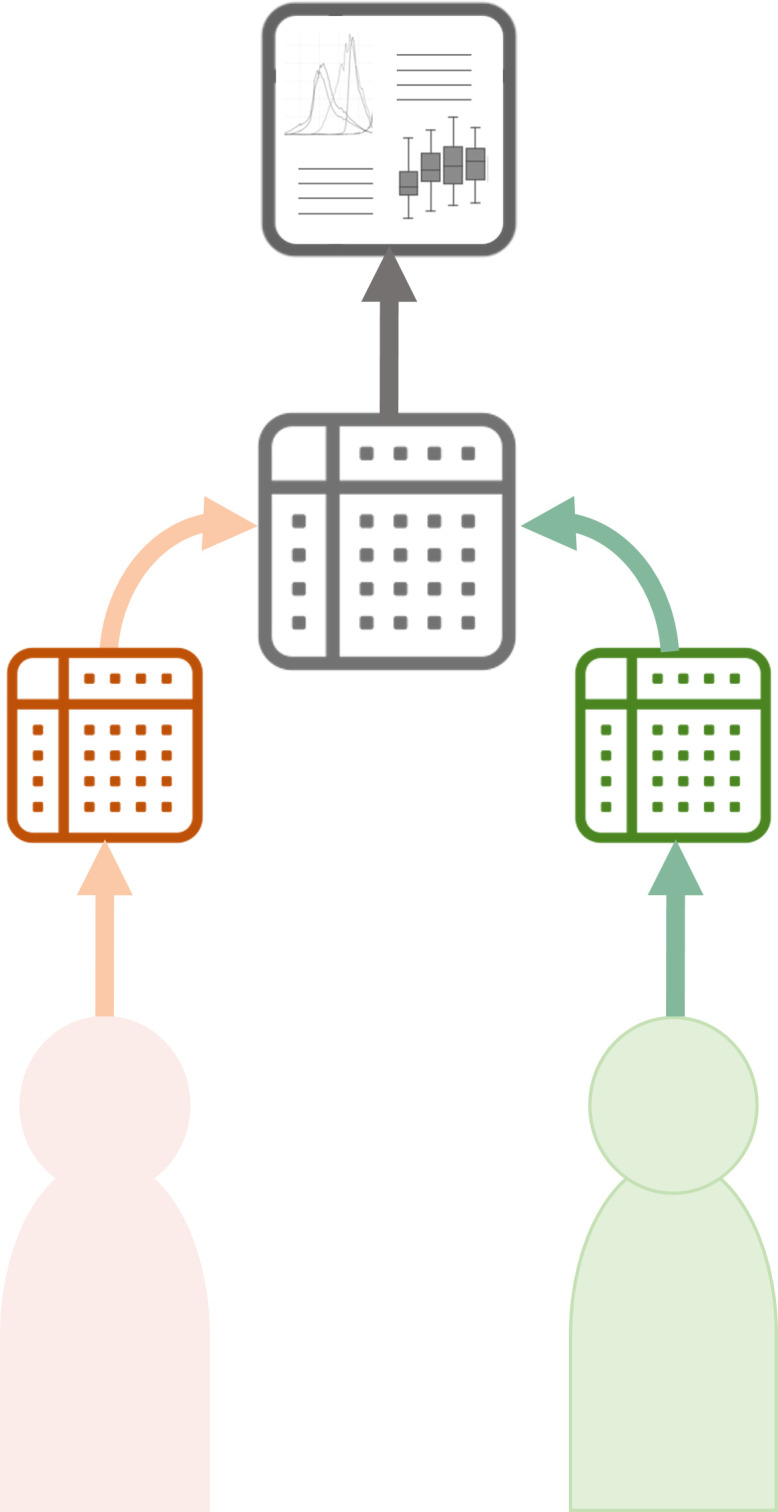
Collaborative meta-analysis. Generators combine their resources and do a joint meta-analysis on the combined dataset. The generators do a joint analysis and generate a collaborative research output.

Whereas direct data sharing results in a unidirectional transfer of data without collaborative opportunities, growing use of data standards has provided greater opportunities to harmonise datasets and combine them for collaborative meta-analysis.^28^ In this model, data generators work together to combine their anonymised datasets and then conduct analyses that provide more statistical power and generalisable findings than when analysing the individual datasets (figure 2). Sometimes in these studies it is also possible to have discovery and validation dataset analysis to measure the generalisability of findings from particular analyses. A significant advantage of collaborative meta-analyses is that the data generators have oversight of the onward use of the data they have generated and can ensure that ethical and informed consent constraints are respected. In addition, they are able to receive recognition for the ongoing analysis of the data they have generated, which can contribute to ensuring sustainability of their research and avoid their work being ‘scooped’ before they have brought it to publication.^29^ This attribution is particularly important in under-resourced research environments where securing research grants is both difficult and also essential for sustainability.

One of the major challenges for performing meta-analyses is the combination of large datasets which may have different data structures and captured variables, even when they are addressing similar primary research questions. The process of data harmonisation and associated data quality checking can be extremely time and resource-consuming, and requires the development of common data models. For example, The International Epidemiology Databases to Evaluate AIDS consortium developed these tools, and used the OMOP common data model (https://ohdsi.github.io/CommonDataModel/index.html) in order to combine international datasets from studies of populations living with HIV/AIDS across multiple countries for meta-analysis and comparative studies.^30^ Large consortia such as the Global Genomic Medicine Collaboration^31^ (https://g2mc.org/), Global Alliance for Genomics and Health^32^ (https://www.ga4gh.org/) and International HundredK+Cohorts Consortium^33^ (https://ihccglobal.org/) now contribute significant resources into developing data standards for wider use, to enable such meta-analyses using health, epidemiological and genomic data without requiring retrofitting and retrospective harmonisation of data for meta-analysis.

### Federated analysis

**Figure 3:**
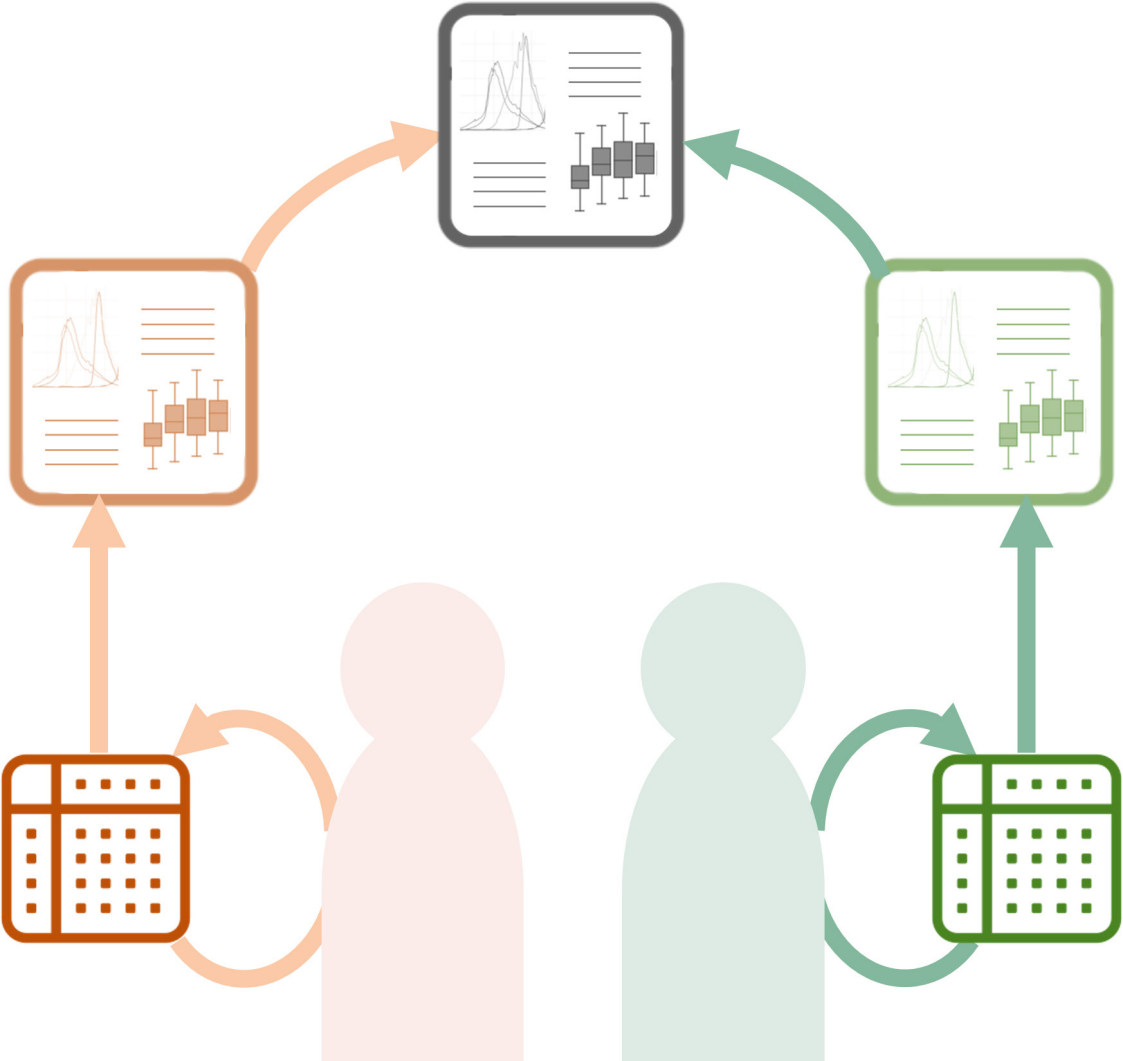
Federated analysis. Researchers independently conduct the same analysis on their own datasets and then combine their analysis outputs. Only the independently generated analysis results are combined in a joint research output.

In some cases, data use permissions, ethical constraints or informed consent limitations mean that data may not be shared with other parties in direct transfer or collaborative meta-analysis agreements. In addition, for very large datasets their size may also prohibit routine transfer of datasets for secondary use. In these cases, federated analysis is another approach that may be used to optimise secondary knowledge generation from datasets that cannot be shared. For the federated data analysis model, datasets are held separately by collaborating parties but are analysed locally in the same way, and then aggregate data and/or findings are combined and reported jointly (figure 3). The complete, granular datasets are never shared and never combined, and the analyses are run by the data generators only on their local dataset. This approach is used increasingly because it can circumnavigate some of the more difficult logistical, procedural and practical challenges that can hinder meta-analyses^34 35^—as described in oncology research using routine health data,^36^ and pharmaco-epidemiology networks,^37^ by way of example.

Similar to collaborative meta-analysis, datasets for federated analysis still need to be comparable so that aggregate results and analysis outputs may be compared and/or combined, but the requirement for an exact replication of data structure and coding is less rigorous, even though federated analysis still requires common data elements and care needs to be taken to ensure that analysis outputs are comparable.^38^ Standardised univariate data exploration of the data from each collaborator can help to flag existing biases in any of the contributory datasets. An additional application of this approach for multicentre collaborative studies is for a single data infrastructure to be created, but with partitioning that allows each centre control of and access to only their own data in the database.^39^ An implementation example for federated database access is the assignment of Data Access Groups in REDCap databases,^40^ ensuring user groups are only able to see certain records in the database that are entered by members of their own user group although the common data structure is used by all. Increasingly, researchers are also practicing federated learning whereby only algorithm weightings are shared and can be integrated by all collaborators in order to build a final model.^41^

### A Trusted Research ecosystem

As data-sharing models evolve, it has become evident that many researchers wishing to undertake secondary analysis on shared datasets will do so in a responsible and considered way, and that trusted and validated users may be able to run their own analyses directly on large datasets under controlled conditions.

#### A Trusted Research Environment

**Figure 4:**
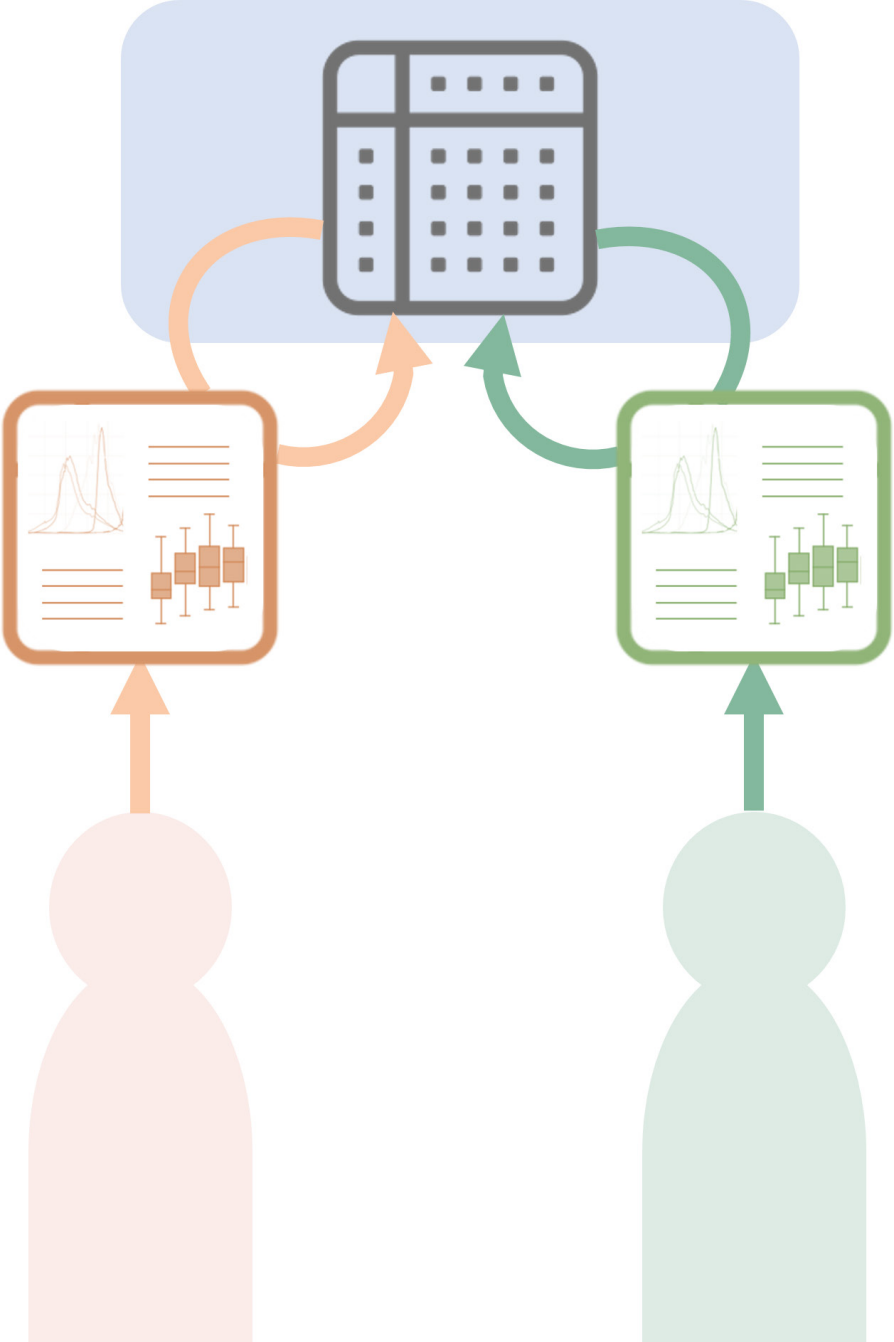
Trusted Research Environment. Researchers register for an account that allows them access to a dataset on a secure platform where they can run analyses and generate outputs, but can only download and take away the outputs of the analyses without copying, downloading or retaining the raw data. Researchers generate independent analyses and research outputs from a common data source.

Generating and managing datasets for sharing can be a time-consuming, labour-intensive task that is often not recognised in assignment of budgets and personnel time. As datasets become ever larger, the number of data consumers wishing to use those data are also rapidly increasing, and many of these are repeatedly requesting related datasets. Organisations holding such large datasets have begun creating platforms where trusted users are able to directly query the complete dataset without visualising the personalised data or extracting any sections of the dataset for download.^42^ In this online environment, the data consumer can run their required data analyses and export only the output and aggregated results (figure 4). This provides the data provider with full control over the access and use of the shared data, while enabling secure access to data for appropriate research purposes. Ongoing query logging tracks the user activities on the platform to ensure accountability. Some examples of TREs include the UK’s Secure Data Environment server (https://digital.nhs.uk/services/secure-data-environment-service) for research access to anonymised health service patient data; the UK Biobank Research Analysis Platform,^43^ the Terra platform developed by the Broad Institute (https://terra.bio/about/mission/) and the Seven Bridges Platform (https://www.sevenbridges.com/platform/).

#### Trusted third party for data linkage, anonymisation and perturbation

Linking datasets from disparate data sources but for the same group of individuals can provide important epidemiological and health insights. In some legislative circumstances, this kind of analysis can only be done using anonymised data which creates the paradox in which identifying data fields are required to perform the linkage, but should not be revealed to the researchers using the linked dataset. In these circumstances, data linkage and subsequent anonymisation and perturbation of the linked dataset may be undertaken by a trusted third party who provides the linkage facility but has no further investment or involvement in the provided and output datasets. This third party will sign a non-disclosure agreement or memorandum of understanding regarding confidentiality, data protection and non-use of the data, as well as committing to deleting all data related to the linkage process within a specified time frame.

## Additional considerations for data sharing

### Considerations for commercial use of data

Use of data for commercial use is a specific use case that comes with many additional and specific complexities. These include IP and potential licensable and/or patentable output. Because of the complexity and the difficulty in generalising these kinds of DSAs, in this study we have focused on sharing for academic research, recognising that the legal and ethical complications of data sharing for commercial purposes require an in-depth review that is beyond the scope of the current analysis.

### Data deidentification and anonymisation

Data deidentification and data anonymisation refer to the processes of preparing, managing and distributing datasets removed of personally identifiable information. This is important in multicentre health research studies, for example, to provide a scalable and secure way for sharing medical information from health service records while safeguarding the privacy of patients.^44^ These approaches can also alleviate concerns that consent requirements for the use of identified data negatively impact research cost, recruitment rates, research duration and outcomes and may also exacerbate recruitment bias (reviewed in^45^,^47^).

Data deidentification is a process used to remove or replace the patient identifiers, such as name and identity number, from private records to prevent the relinking of the personally identifiable data to the data subject.^44^ At the earliest opportunity, personal identifiers of the medical data are encrypted and the deidentified dataset is stored in a separate database. An internal anonymous key is used to link the deidentified data with the attribute data, and the dataset is always differentially perturbated for each dataset release to prevent linkage of independent datasets released leading to reidentification. Necessary access to databases with personally identifiable information, for example by database developers or analysts undertaking data linkage or curation, is tightly managed and restricted to absolute instances and subjected to both specific approvals and governance undertakings. Such data deidentification still allows for the future reassociation between the personally identifiable data and the individual, and similarly pseudonymisation replaces personally identifiable information with pseudonyms with a separate lookup table that can map pseudonyms to personally identifiable information. True data anonymisation, however, removes this association while preserving the utility of the information as much as possible.

Data perturbation is the addition of alterations or noise to the data to prevent the reidentification of the study participants and can be applied on different types of datasets to protect both privacy and confidentiality, including analysis extracts, research extracts without informed consent, and data in databases that are used for maintenance and development work within data storage environments.^48^ Some examples of simple types of data perturbation include using only year of birth rather than date of birth; adding an undisclosed integer to all event dates so that times between key dates remain unchanged but reidentification through date-defined events is minimised, and using age in years at an event rather than providing birth year or event dates. More advanced statistical approaches may also be used to ensure privacy, providing a framework for ensuring that it is very difficult to infer information about individuals from a dataset while ensuring results of analyses remain true to the underlying dataset.^49 50^ Anonymisation techniques are vulnerable to reidentification attacks using auxiliary datasets to compromise the privacy of data subjects, and it is advisable to apply perturbation to as many fields as possible for all requests, with perturbation techniques varying per request depending on the intended research questions and ethical concerns. Techniques and metrics such as k-anonymisation and metrics such as l-diversity can be used to reduce these risks.^44 51^

## Data-sharing agreements

A DSA is a formal document which allows for the regulation of data exchange between data generators and consumers in a controlled manner. This is done by defining a priori specific guidelines and procedures agreed on by both parties on what is required, permissible or denied with respect to data covered by the agreement.^52^ While there are multiple clauses in a DSA (table 2), in this paper we have focused on informed consent, benefit sharing, IP, intended outputs and authorship, attributions and acknowledgements as they are central to ensuring ethical and equitable data sharing among researchers, and can often be focal points for contention if they are not established up front. Online supplemental table S1 provides descriptions of some of the most commonly included DSA elements.

**Table 2:** Data sharing agreement modules for four types of data sharing

Type of data sharing	Direct share	Collaborative	Federated	TRE
Agreement module				
Data generator details	x	x	x	x
Roles and responsibilities	x	x	x	x
Data consumer details	x	x	x	x
Data consumer roles and responsibilities	x	x	x	x
Purpose of sharing initiative	x	x	x	x
Anticipated or intended output	x	x	x	x
Appropriate ethics and consent documents	x	x	x	x
Term/duration of sharing initiative	x	x	x	x
IP ownership	x	x	x	x
Data ownership	x	x	x	x
Description of data and/or biospecimens	x	x	x	x
Mode of data transfer (including costs)	x	x		
Mode of biospecimen transfer (including costs)	x	x		
Mode of sharing data analysis code		x	x	
Termination of sharing initiative	x	x	x	x
Timeline for retention of data and/or biospecimen	x	x		
Procedure for permanent deletion of data	x	x		
Procedure for discarding of biospecimen	x	x		
Risk assessment	x	x	x	x
Type of benefit sharing	x	x	x	x
Authorship for publications	x	x	x	x
Acknowledgement statement	x	x	x	x

IP, intellectual property; TRE, trusted research environment.

### Informed consent

Consent protocols and documents must be robust, and those conducting the primary study need to ensure that relevant consent that allows for secondary use of the data is in place, and that the consent documents are aligned with the intended onward use of the data and/or biospecimens.^53^ This is essential for direct sharing where control over secondary use is completely relinquished and the primary data generators lose oversight of the onward use of the data. In addition, if any commercial onward use is intended, participants need to have specifically consented for the use of their data for commercial purposes, and any share in profits or benefits from such onward use, or lack thereof, needs to be clearly identified in the consent information for the participants.

### Benefit sharing

Generating primary data in under-resourced settings is often a challenging and expensive undertaking for researchers, and participation in research may itself be challenging in under-resourced environments. There is, therefore, an ethical imperative to ensure maximal return of benefits from onward sharing of these data. Such benefit sharing is often overlooked, especially for secondary use of data. While collaborative and federated sharing and the use of TREs ensure that the primary data generators are still involved in the secondary use of data, in the case of direct sharing consideration should be given to ensuring both the data generators and research participants might benefit from the secondary use of their data. While benefit sharing is not yet commonly inculcated in research planning, there is increasing recognition of the need to plan for benefit sharing, and available frameworks provide guidance for implementation.^54^ For non-human genetic resources, such benefit sharing is governed by the access and benefit-sharing provisions of the United Nations’ Convention on Biological Diversity and its supplementary Nagoya Protocol. These agreements recognise that countries have the sovereign right to regulate access to their genetic resources. It is uncertain whether digital sequence information (DSI) associated with those genetic resources are included in these agreements, but since December 2022, processes are underway to incorporate DSI in the benefit-sharing accords.^55^ It is also important to recognise that benefit-sharing protocols may need to be tailored according to the specific requirements of under-represented population groups.^15 56^

### Intellectual property

For all modes of data sharing, ownership of the current and future IP rights associated with the shared data must be clearly assigned, and this should be done a priori to avoid problems arising in the future. In addition, as this has legal implications this element of DSAs should be compiled with the input of legal and/or technology transfer departments at the institutions of the parties entering into the DSA.

### Intended output

A detailed description of the intended or anticipated outputs from the shared data, such as manuscripts, training materials, tools and products, needs to be described in detail a priori to ensure that they align with the consent provided by participants, especially in the case of direct sharing where control of the data is relinquished by the data producers and they lose oversight of onward data use. Having clearly defined outputs can also strengthen collaborative and federated analysis, by ensuring that the parties involved are working towards explicit common goals, and that they also do not accidentally infringe on each other’s independent research agendas.

### Authorship, attribution and acknowledgements

Agreement on the authorship and attribution plan for outputs generated from the data and/or biospecimens should be reached and recorded in DSAs. For academic output, documenting first, senior and corresponding authorship in future publications arising from the agreed data sharing can focus collaborators’ roles and prevent disputes down the line. For direct sharing, the data generators should also be adequately acknowledged in research outputs emanating from the secondary use of the data that they have made available. In collaborative and federated DSAs, this can ensure that such agreements do not disadvantage any of the partners, and ensuring this kind of equity is especially important where partnerships occur between more established and early career researchers, between highly resourced and poorly resourced research groups, and between researchers in the global North and global South.^57^ To foster equity in research, there is increasing recognition of the need to encourage primary data generators from under-resourced environments and the global South to take active roles in subsequent research using the datasets they generated, and to take on senior author roles in subsequent publications.

## Conclusion

The value to be derived from secondary use of data and biospecimens is undisputed. It is more complex, however, to ensure that such secondary use of resources is done in a way that is ethically sound and respects the preferences and privacy of the participants who donated those resources for research. In addition, there is increasing awareness of the need for equitable research agreements that do not reinforce the inequitable research dynamics that have been common to date.

Here, we have described four different modes of data sharing that may provide ways for ethical secondary use of data, including ways of sharing that might be used where data cannot be directly shared onwards to third parties. This need arises most frequently in situations where data are particularly sensitive, where informed consent for secondary analysis has not been provided by research participants, and for legacy datasets for which terms of consent were insufficient or not documented. While direct, unidirectional sharing has been the most common mode of sharing to date, with increasingly granular health and personal data the risks to individual participants of reidentification and breach of privacy are also increasing. We have outlined here some of the approaches used for data deidentification, anonymisation and perturbation, which all increase the security of participants when their data are shared onward for secondary analyses. As the global repositories of granular personal data rapidly expand, the availability of data to triangulate for reidentification of individuals also increases along with the risk of data breach, with the consequence that these approaches to prevent reidentification by anonymisation and perturbation are more important than ever before.

We anticipate that the development of more nuanced data-sharing models such as those described here may facilitate DSAs which might not have previously been possible. For example, a common concern of data generators is that they do not wish to lose oversight of how the data they have collected from participants are being used by other researchers; and another is that data generators, especially those with fewer resources for data analysis, might be scooped by better resourced research groups as soon as they make their data resources available. The options for collaborative meta-analysis and federated analysis both provide models in which these concerns are fully addressed without hindering the possibility of onward use of data resources. Concerns about data privacy, the potential for misuse of sensitive data and risks to participant privacy may also be taken into consideration through federated analysis or the use of a TRE. These data-sharing solutions do not require centralisation of data and provide opportunities to negotiate collaborative secondary research and benefit-sharing, and we have also provided an overview of the types of clauses which should be included in DSAs using these approaches.

We have, in this way, approached the challenges for secondary sharing of sensitive health data with a solutions-based lens, proposing different models of data sharing that can overcome common barriers to secondary analysis. While the approaches we have described are not exhaustive, we hope to encourage creative thinking that moves beyond direct, unidirectional sharing for secondary use, and to facilitate collaborative and equitable data sharing that can effectively advance and support a growing evidence base for the provision of optimal healthcare.

## Supplementary material

10.1136/bmjgh-2023-013092 online supplemental file 1

## Data Availability

Data sharing not applicable as no datasets generated and/or analysed for this study.
