# Supplementary material for: Multiple modes of data sharing can facilitate secondary use of sensitive health data for research
Source: BMJ Glob Health. 2023 Oct 6;8(10):e013092. doi: 10.1136/bmjgh-2023-013092 (PMC10565310; doi:10.1136/bmjgh-2023-013092)
Supplement: online supplemental file 1 [file bmjgh-2023-013092supp001.pdf]

Supplementary Data: Table S1. Description of elements to be included in Data Sharing Agreements

Data Sharing Agreement Element	Description
Type of agreement	The type of data sharing agreement being entered into (direct share, collaborative, federated or trusted research environment). Define intended commercial use explicitly.
Participant details	Full names, contact details and institutional affiliations of the data sharers and data receivers
Roles and responsibilities	What is expected of the different individuals and entities that are part of the agreement. This can include who has signing rights, the primary contact, restrictions
Third party participant details	Full names, contact details and institutional affiliations of any third party individuals or organisations that will have access to the data and/or biospecimen (e.g. commercial laboratory for sample processing)
Third party roles and responsibilities	What is expected of the different individuals and entities that are part of the agreement. This can include who has signing rights, the primary contact, restrictions
Purpose of sharing initiative	What will the shared data/biospecimen be used for. Specific aims clearly explaining the onward use of the data/biospecimen
Intended /anticipated output	Clear description of the intended/anticipated output from the shared data/biospecimen. E.g. manuscripts, genotype/WGS data from DNA, a new tool
Appropriate Ethics Review Board documentation	Where appropriate (ie. samples and data of animal or human origin) Proof of Ethics Review Board approval for the original study; proof of Ethics Review Board approval for intended secondary use study.
Appropriate consent documents	Availability of appropriate informed consent templates that were used in the original study, to confirm that the informed consent provided by participants is aligned with the aims of the onward use of the data/biospecimen.
Duration of sharing initiative	How long the sharing initiative will be in place (calendar time) i.e. commencement and end dates
IP ownership	Who will be the owners of any intellectual property generated using the data/biospecimens
Ownership	Who owns the current and future associated data/biospecimens
Description of data and/or biospecimen to be shared	Detailed description of the data/biospecimens including type and quantity e.g. 80 gb of unprocessed micro-array genotype data; 400 samples of human DNA
Mode of data transfer (including costs)	Detailed description of how data will be transferred between the parties in the agreement, including information about secure data transfer platforms, encryption protocols and password protection plans.
Data storage	Detailed description of how data will be stored securely e.g. cloud, institutional servers. Also include version control plans, backup plans and, if appropriate, disaster recovery plans

Mode of biospecimen transfer (including costs)	Detailed description of how biospecimen will be transferred between the parties including shipping conditions and who will cover the associated costs
Mode of sharing data analysis code	Description and links to code sharing repositories e.g Github
Termination of sharing initiative	Detailed description of what happens at the end of the sharing initiative including any formal documents which might need to be signed to effect the termination at the end of the agreed timelines. Conditions under which the sharing initiative can be terminated by either party before the agreed timelines.
Timeline for retention shared data and/or biospecimen	How long data and/or biospecimen can be kept by those receiving them (calendar time)
Procedure for permanent deletion of data	Conditions under which data can be permanently deleted
Procedure for discarding of biospecimen	Detailed description of how and when biospecimen can be permanently discarded by either party. This should include a time frame, the appropriate bio-safety procedure and any reporting requirements for discarding the biospecimens.
Risk assessment	Detailed description of the risks associated with sharing the data or biospecimens. These include risks to the participants and to the data generators: Risks to participants can include and are not limited to re-identification and/or loss of privacy and confidentiality, social risks such as stigma and economic risks such as change in insurability. Risks to researchers can include and are not limited to career risks due to association with certain research and financial risks associated with the cost of running a project.
Type of benefit sharing	Description of what kind of benefit sharing will be undertaken by the recipients of the data/biospecimens
Authorship for publications	Detailed agreement on the authorship of output generated from the data and/or biospecimen in particular first and senior authorship
Acknowledgement statement	An agreed Acknowledgement statement regarding the source of data/biospecimens and acknowledging funding sources
